# Supplementary material for: The First Evidence on the Occurrence of Bisphenol Analogues in the Aqueous Humor of Patients Undergoing Cataract Surgery
Source: J Clin Med. 2022 Oct 29;11(21):6402. doi: 10.3390/jcm11216402 (PMC9655480; doi:10.3390/jcm11216402)
Supplement: Supplementary file 1 [file jcm-11-06402-s001.zip › jcm-1847726-supplementary.pdf]

	Mean concentration [ng mL^{-1}]; n=3						coexisting disease	
Patient number	BPA-1	BPA-2	BPA-free	ΣBPs	Age	Gender	ocular pathology	other pathology: diabetes
KB 31	0.5	0.53	<LOD	1.03	91	w	-	-
MZ 41	3.48	2.96	<LOD	6.44	81	w	-	-
SZW 4706	<LOD	<LOD	<LOD	0	75	m	AMD	+
KAJ 5609	<LOD	<LOD	<LOD	0	66	w	-	-
DW 44	1.2	1.66	<LOD	2.86	78	m	AMD	-
RB 53	1.18	2.19	<LOD	3.37	69	w	-	-
PIH 3703	<LOD	<LOD	<LOD	0	85	w	glaucoma	-
KRM 4601	<LOD	<LOD	<LOD	0	76	w	-	-
DA 64	1.04	1.13	0.35	2.52	68	w	AMD	-
GJ 35	2.28	2.45	<LOD	4.73	87	w	-	-
FW 69	1.86	2.58	<LOD	4.44	53	m	-	-
KOB 3001	<LOD	<LOD	<LOD	0	92	w	glaucoma	-
ZJ 45	<LOD	<LOD	<LOD	0	77	m	-	-
WE 54	<LOD	<LOD	<LOD	0	68	w	glaucoma	-
SZW 4003	<LOD	<LOD	<LOD	0	82	w	-	-
TZ 51	2.65	3.82	1.57	8.04	71	m	AMD	-
ST 40	<LOD	<LOD	0.5	0.5	82	w	-	-
KJ 36	<LOD	<LOD	0.6	0.6	86	w	-	-
WH 46	<LOD	<LOD	0.3	0.3	76	m	glaucoma	-
SM 46	<LOD	<LOD	0.32	0.32	76	w	-	-
CJ 40	<LOD	<LOD	0.98	0.98	82	w	AMD	-
KOK 3704	<LOD	<LOD	<LOD	<LOD	85	w	AMD	-
IWS 4610	<LOD	<LOD	<LOD	<LOD	76	w	AMD	-
ZAP 5110	<LOD	<LOD	<LOD	<LOD	71	m	-	+
KS 59	1.85	2.78	1.69	6.32	63	m	-	-
IWS 46 10	2.61	<LOD	<LOD	2.61	76	w	-	-
PIJ 3207	<LOD	1.9	<LOD	1.9	90	m	-	-
KUM7312	0.84	<LOD	<LOD	0.84	49	w	AMD	-
KUS 5107	3.8	3.2	<LOD	7	71	m	-	-
KRW4501	<LOD	<LOD	8.65	8.65	77	m	-	-
STT 5201	<LOD	0.9	<LOD	0.9	70	m	AMD	-
LAZ 5304	<LOD	<LOD	0.3	0.3	69	w	-	-
POH 4508	<LOD	<LOD	<LOD	0	77	w	-	-
BIS 4505	<LOD	<LOD	1.2	1.2	77	m	-	-
KAA 4507	7.51	6.87	<LOD	14.38	77	w	-	-
DĄJ 5710	1.54	1.23	<LOD	2.77	65	m	-	-
RA 46	0.42	0.54	<LOD	0.96	76	w	-	+
MS 48	3.76	3.91	<LOD	7.67	74	m	-	+

CM 44	1.12	1.39	<LOD	2.51	78	m	-	+
DĄU 4701	1.12	0.4	0.51	2.03	75	w	-	+
BUB 3605	<LOD	<LOD	<LOD	0	86	w	-	+
DUS 3603	<LOD	<LOD	<LOD	0	86	m	glaucoma	-
ŚWA 3706	<LOD	<LOD	<LOD	0	85	w	AMD	-
ZDH 4701	<LOD	<LOD	<LOD	0	75	m	-	-

Table S1 The raw data for the male (m)/female (w) cataract individuals with and/or without coexisting ophthalmic pathology (AMD, glaucoma) presented by mean concentration [ng mL-1] of BPA-1, BPA-2, BPA-free, and ΣBPS. The measurements of BPs concentration were performed by UHPLC-MS/MS. Conditions of measurements were described in section 2.4.
